# Purification and biochemical characterization of pancreatic phospholipase A2 from the common stingray Dasyatis pastinaca

**DOI:** 10.1186/1476-511X-10-32

**Published:** 2011-02-17

**Authors:** Abir Ben Bacha, Aida Karray, Emna Bouchaala, Youssef Gargouri, Yassine Ben Ali

**Affiliations:** 1 Laboratoire de Biochimie et de Génie Enzymatique des Lipases, ENIS route de Soukra, BP1173, University of Sfax - 3038 Sfax, Tunisia

## Abstract

**Background:**

Mammalian sPLA2-IB are well characterized. In contrast, much less is known about aquatic ones. The aquatic world contains a wide variety of living species and, hence represents a great potential for discovering new lipolytic enzymes.

**Results:**

A marine stingray phospholipase A_2 _(SPLA2) was purified from delipidated pancreas. Purified SPLA2, which is not glycosylated protein, was found to be monomeric protein with a molecular mass of 14 kDa. A specific activity of 750 U/mg for purified SPLA2 was measured at optimal conditions (pH 8.5 and 40 °C) in the presence of 4 mM NaTDC and 8 mM CaCl_2 _using PC as substrate. The sequence of the first twenty first amino-acid residues at the N-terminal extremity of SPLA2 was determined and shows a close similarity with known mammal and bird pancreatic secreted phospholipases A2. SPLA2 stability in the presence of organic solvents, as well as in acidic and alkaline pH and at high temperature makes it a good candidate for its application in food industry.

**Conclusions:**

SPLA2 has several advantageous features for industrial applications. Stability of SPLA2 in the presence of organic solvents, and its tolerance to high temperatures, basic and acidic pH, makes it a good candidate for application in food industry to treat phospholipid-rich industrial effluents, or to synthesize useful chemical compounds.

## Background

Phospholipases A2 (PLA2) comprise a set of extracellular and intracellular enzymes that catalyze the hydrolysis of the sn-2 fatty acyl bond of phospholipids to yield fatty acids and lysophospholipids [1]. The intracellular PLA2 s are divided into cPLA2 (cytosolic calcium dependent, group IV) and iPLA2 (cytosolic calcium independent, group VI), based on the Ca^2+ ^requirements needed for basal activity. cPLA2 requires micromolar Ca^2+ ^for membrane translocation but not for catalysis, possesses a preference for phospholipids containing AA, and have high molecular mass (> 60 kDa). iPLA2 exhibits no substrate specificity for AA-containing phospholipids and no Ca^2+ ^requirement for activity and has high molecular mass (about 85 kDa) [1-3]. The extracellular (secreted) PLA2 s (sPLA2) have low molecular masses (13-18 kDa), require millimolar calcium concentrations for catalytic activity, and do not manifest significant fatty acid selectivity in vitro. To date, 11 forms of mammal sPLA2 have been identified and classified according to their origin, sequence similarity and molecular mass as well as substrate specificity into groups IB, IIA, IIC, IID, IIE, IIF, V, X, III, XIIA and XIIB [4,5]. There is also a class of PLA2 s called platelet-activating factor (PAF) acetylhydrolases [6].

sPLA2-IB is also known as the pancreatic-type PLA2. It is synthesized by the pancreatic acinar cells, and after secretion as a zymogen into the pancreatic juice, an N-terminal heptapeptide of the inactive zymogen is cleaved by trypsin to yield an active enzyme in the duodenum.

sPLA2-IB is also highly expressed in the stomach and is present at lower levels in lung, spleen, liver, colon and eyes [7-9]. Receptors for this enzyme have been identified in various tissues, and group IB PLA2 is now reported to play a role in cell proliferation and hormone release via these receptors in non-digestive tissues [7,10,11]. These findings reveal the physiological importance of group IB PLA2 in non-digestive tissues, in addition to digestive lipolysis in the intestinal tract.

Cartilaginous fish, represented by sharks, skates and rays, are generally considered as the most primitive living jawed vertebrates. They first appeared during the Ordovician period about 450 million years ago sharing a common ancestor with a jawed vertebrate ancestor, placoderm. The extinction of placoderm at Devonian-Carboniferous boundary makes cartilaginous fish the oldest taxa of extant jawed vertebrates, pushing them to the edge of jawless-jawed transition. To date, the cartilaginous fish are also the oldest vertebrates possessing a complex digestif system like mammals [12].

Mammalian sPLA2-IB are well characterized [13-20] and recently some studies are carried on bird PLA2 [21-23]. In the contrast, much less is known about aquatic ones [24-30]. The aquatic world contains a wide variety of living species and, hence represents a great potential for discovering new enzymes. It is therefore interesting to study some catalytic and biochemical properties of a purified marine PLA2 to gain more insights into their action mode on phospholipids. This paper reports, for the first time, the purification of phospholipase A2 from the same organ. This phospholipase tentatively named stingray pancreatic phospholipase A2 (SPLA2) was characterized using the emulsified system.

## Results

### Activation of SPLA2 by trypsin

**Figure 1:**
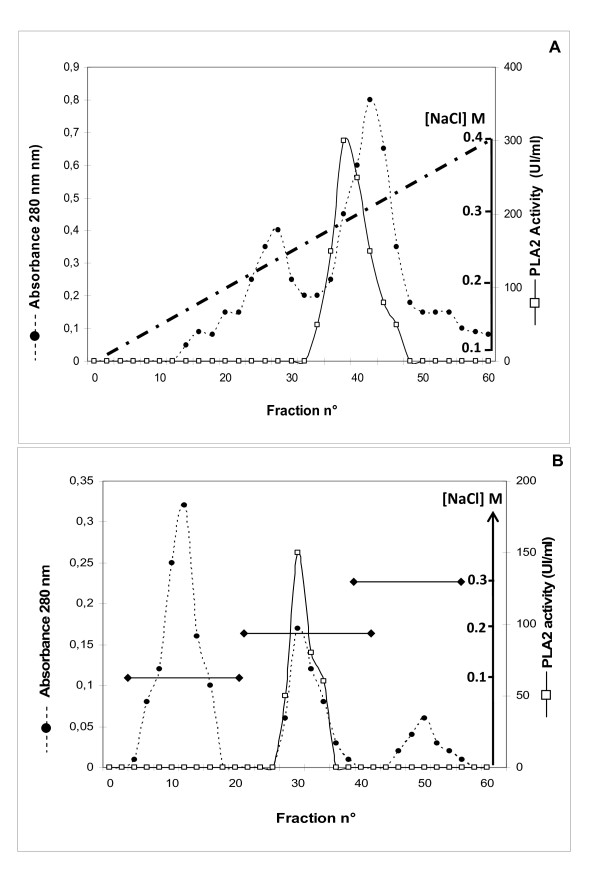
**Chromatography of stingray PLA2 on FPLC Mono-S Sepharose and Mono-Q Sepharose**. (A) Chromatography of stingray PLA2 on FPLC Mono-S Sepharose. The column (2.6 cm × 20 cm) was equilibrated with 100 mM acetate buffer, pH 4.5, containing 0.05% Triton X-100 and 2 mM benzamidine (buffer A); a linear salt gradient (0.1 to 0.4 M NaCl) in buffer A was applied to the column; gradient chamber 100 ml; 2 ml fraction; flow rate, 30 ml/h. (B) Chromatography of stingray PLA2 on Mono-Q Sepharose step. The column (1.5 cm × 20 cm) was equilibrated with 25 mM tris-HCl buffer, pH 8, containing 25 mM NaCl. Proteins were eluted by three washes applied to the column (3 × 80 ml from 100 mM to 300 mM NaCl). The flow rate was 40 ml/h and the fraction size was 4 ml. SPLA2 activity was measured as described in materials and methods using PC as substrate. Active fractions (28 to 34) were gathered.

No phospholipase activity was detected in freshly crude extract of delipidated pancreas of stingray using PC emulsions (Figure 1). The maximum PLA2 activity was obtained after incubation at room temperature during 40 min, PLA2 activity did not increase when exogenous trypsin was added at different ratios to the homogenate solution then endogenous proteases are sufficient to achieve PLA2 activation (data not shown).

It has emerged from several kinetic studies on phospholipases A_2 _that the N-terminal propeptide may play an important role in the expression of the maximum catalytic activity measured in vitro [31].

The most illustrative example was reported by de Haas' group on pancreatic phospholipase A_2_, which is known to be secreted by the pancreas as a zymogen which is highly active on water soluble short chain phospholipids, but not able to hydrolyse long chain phospholipids present at the interface. Limited proteolysis by trypsin of the Arg7-Ala8 peptide bond transforms the inactive zymogen into an active enzyme [32].

### Purification of SPLA2 from the stingray pancreas

20 grams of delipidated powder of the stingray pancreas was suspended in 300 ml 50 mM Tris-HCl buffer, pH 8.5, containing 0.05% Triton X-100 and 150 mM NaCl (buffer A) and ground mechanically twice for 30 s using the Waring Blendor system. The mixture was stirred with a magnetic bar for 45 min at room temperature and then centrifuged for 30 min at 12,000 rpm. The supernatant contained 450 PLA2 units per gram of delipidated pancreatic tissue.

#### - Heat and acidic treatment

In contrast to marine snail (mSDPL) and crab (CDPL) digestive phospholipases purified recently in our laboratory [28,30], SPLA2 present in the homogenate can tolerate, without any denaturation, the incubation at high temperature. The stingray extract PLA2 solution was incubated 15 min at 65 °C. After rapid cooling, insoluble denatured proteins were removed by centrifugation during 30 min at 12,000 rpm. Afterward, the pH of the previous supernatant was brought to 3.0 by adding 6 N HCl under gentle stirring at 0°C. After centrifugation (30 min at 12,000 rpm), the clear supernatant, which was adjusted to pH 7 with 6 N NaOH, contained 85% of starting amount of PLA2.

#### - Ammonium sulfate precipitation

The treated supernatant (250 ml, 7650 U) was brought to 70% saturation with solid ammonium sulphate under stirring conditions and maintained during 45 min at 4 °C. After centrifugation (30 min at 12.000 rpm), the precipitated PLA2 was resuspended in 10 ml of buffer A containing 2 mM benzamidine. Insoluble material was removed by centrifugation during 10 min at 12.000 rpm. Approximately 68% of the starting amount of PLA2 was recovered.

#### - **Ethanol fractionation**

An equal volume of pure ethanol solution was added to the supernatant (10 ml, 6120 U) at 0 °C. Precipitated proteins were removed by centrifugation and the supernatant was added slowly with four times its volume of ethanol to bring the alcohol concentration to 90% (v/v) at 0 °C. After centrifugation for 30 min at 12.000 rpm the ethanol precipitated PLA2, which contains about 50% of the enzyme starting amount, was solubilized in of 100 mM acetate buffer pH 4.5 containing 0.05% TX-100 and 2 mM benzamidine (buffer B). In the present study, we found this step critical to eliminate the last traces of lipids facilitating the filtration chromatography step.

#### Filtration on Sephadex G-50

The PLA2 sample was submitted to gel filtration through a Sephadex G-50 column (95 cm × 2.6 cm) equilibrated with buffer B. Elution of proteins was performed with the same buffer at 30 ml/h. The fractions containing the PLA2 activity eluted between 1.5 and 2 void volumes were pooled together (data not shown).

#### - **FPLC cation exchange Mono-S Sepharose**

The pooled active fractions of Sephadex G-50 column were applied to a Mono-S column (2.6 cm × 20 cm) equilibrated with buffer B. Non fixed proteins were washed out with 0.1 M NaCl in buffer B. The elution of the adsorbed proteins was then performed with a linear gradient of NaCl (0.1 to 0.4 M). As shown in the elution diagram, SPLA2 activity emerged in a single peak (Figure 1A) at 0.27 M NaCl. The fractions of this peak were pooled, lyophilized and then dialyzed over night at 4 °C against 25 mM Tris HCl buffer pH 8 containing 25 mM NaCl and 2 mM benzamidine (buffer C). The recovery of PLA2 from Mono-S column was of about 45% of the starting amount of the enzyme.

#### - Anion exchange chromatography

Dialyzed active fractions were subjected to anion-exchange chromatography using a Mono-Q column (1.5 cm × 20 cm) equilibrated with buffer. The column was rinsed with 100 ml of buffer C containing 100 mM NaCl allowed to eliminate a first peak with high absorbance. SPLA2 was eluted from Mono-Q Sepharose upon a single wash with the same buffer containing 200 mM NaCl. One peak was then obtained and only 8 fractions containing pure SPLA2 were pooled (figure 1B). Active fractions were pooled and lyophilized. At this stage of purification, the enzyme presented a specific activity of 550 U/mg.

#### - RP-HPLC C-8 column

**Figure 2:**
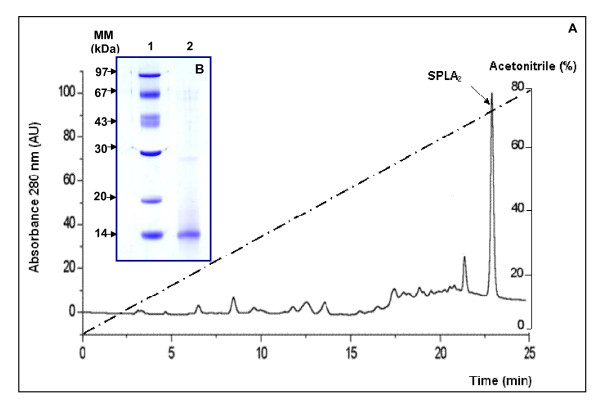
**RP-HPLC column and SDS-PAGE (15%) of SPLA2**. (A) RP-HPLC on a eurospher 100, C-8 column, elution was performed at room temperature within 30 min using a gradient from 0 to 80% solvent B at a flow rate of 1 ml/min. [solvent A, water/trifluoroacetic acid (1000:1, v/v) and solvent B, acetonitrile]. The effluent was monitored at 280 nm. The gradient is indicated by the dotted line. AU: Arbitrary Units. (B) SDS-PAGE (15%) of pure SPLA_2_. Lane1, molecular mass markers (Pharmacia); lane 2, 7 μg of SPLA2 eluted from RP-HPLC. The gel was stained with Comassie blue to reveal proteins.

Thirty units of lyophilized sample from Mono-Q column were applied to RP-HPLC eurospher 100, C-8 column (250 mm × 4.6 mm). PLA2 activity was detected in a fraction eluted at 70% acetonitrile as a single peak (Figure 2A) and the overall recovery of the enzyme activity was 23% of the starting amount.

The purification flow sheet given in Table 1 shows that the specific activity of pure SPLA2 reached 750 U/mg, when PC or egg yolk emulsions were used as substrates at pH 8.5, 40 °C and in the presence of 4 mM NaTDC and 8 mM CaCl_2_. The fractions containing the SPLA2 activity were pooled and analysed on SDS-PAGE (Figure 2B). This figure shows that SPLA2 is homogenously pure and has an apparent molecular mass of 14 kDa. This result was in line with the molecular mass determined under native conditions, using gel filtration on FPLC column Superdex 75 (1 × 30 cm) (data not shown). These data suggested that SPLA2 was a monomeric protein like all the sPLA2 described in previous works [13-23].

**Table 1:** Flow sheet of stingray PLA2 purification.

Purification step	Total ^(a) ^activity (units)	Protein ^(b)^ (mg)	Specific activity (U/mg)	Activity recovery (%)	Purification factor
**Extraction**	9000	3500	2.6	100	1

**Heat and acidic treatment**	7650	1050	7.3	72	2.8

**(NH_4_)_2_SO_4_** **Precipitation** **(30-70%)**	6120	500	12.3	68	4.7

**Ethanol fractionation** **(50-90%)**	5250	150	35	58	13.46

**Mono-S** **Sepharose**	4050	8.1	250	45	96

**Mono-Q** **Sepharose**	2900	5.3	550	32	211.5

**RP-HPLC**	2100	2.8	750	23	288.5

(a) 1 Unit: μmole of fatty acid released per min using PC emulsion as substrate in the presence of 4 mM NaTDC and in the presence of 8 mM CaCl_2_.

(b) Proteins were estimated by Bradford method (32). The experiments were conducted three times.

The presence of glycan chains in pure SPLA2 was checked. Our results showed that the aquatic PLA2, like mammalian and bird PLA2, is not glycosylated (data not shown) [20-23].

### NH_2_-terminal sequencing of SPLA2

Purified SPLA2 was denaturated, reduced and alkylated as described in Section 2 and dialysed against distilled water. The NH_2_-terminal sequencing of the PVDF transferred band from an electrophoretic gel allowed unambiguously the identification of the twenty first N-terminal residues of SPLA2. The same sequences were obtained when the pure SPLA2 was transferred without alkylation on a PVDF membrane. Result presented in Table 2 shows the N-terminal sequence, of SPLA2, together with those of dromedary [20], turkey [21], ostrich [22] and chicken [23] PLA2. N-terminal sequence of marine PLA2 exhibits a high degree of homology with N-terminal sequences of mammal and bird ones. However, no similarity of the CDPL [28] and mSDL [30] N-terminal amino acid sequences with known digestive phospholipases was found.

**Table 2:** Alignment of the N-terminal sequence of SPLA2 with chicken, turkey, ostrich and dromedary PLA2.

	**1**	**5**	**10**	**15**	**20**	
**Stingray**	**:AIFEF**	**RSMIK**	**CTIPP**	**SSPIL**	**D**	**This study**
**Chicken**	**:A**LWEF	**R**S**MIK**	**C**A**IP**H	**S**HPF**L**	E	(23)
**Ostrich**	**:A**VWQF	**R**E**MIK**	**C**T**IP**P	**S**DDL**L**	D	(22)
**Dromedary**	:**A**LWQF	**R**D**MIK**	**C**K**IP**D	**S**SPL**L**	D	(20)
**Turkey**	**:A**LFEF	**R**S**MIK**	**C**T**IP**G	**S**DPE**L**	D	(21)

Residues in bold indicate identical amino acids.

### Enzymatic properties of the purified SPLA2

#### Effect of temperature on phospholipase activity and stability

**Figure 3:**
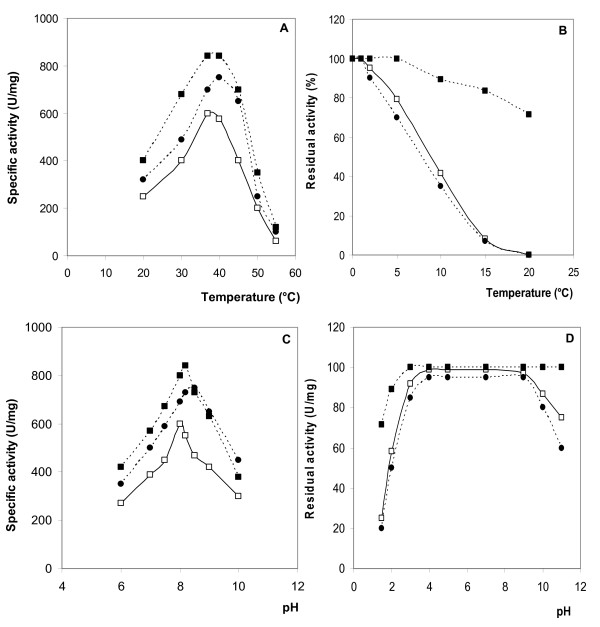
**Effect of temperature and pH**. (A) Effect of temperature on SPLA2, OPLA2 and DrPLA2 activity. (B) Effect of temperature on SPLA2, OPLA2 and DrPLA2 stability. The enzyme activity was tested at various temperatures using PC as substrate in the presence of 10 mM Ca ^2+ ^and 4 mM NaDC and at standard conditions after incubation at 70°C at different times. (C) Effects of pH on SPLA2, OPLA2 and DrPLA2 activity and stability. (D) Effects of pH on SPLA2, OPLA2 and DrPLA2 activity and stability. The enzyme activity was tested at various pH using PC as substrate in the presence of 10 mM Ca^2+ ^and 4 mM NaDC and at standard conditions after incubation at different pH. All experiments were repeated at least three times. SPLA2 (black circle); DrPLA2 (black square); OPLA2 (open square).

Phospholipase activity was tested at temperatures ranging from 20 to 55 °C using homogeneous PC emulsion as substrate (figure 3A). For the sake of comparison we also report the results for dromedary and ostrich pancreatic phospholipases in Figure 2. The maximal SPLA2 activity was measured at 40 °C. This optimum was similar to that of mammal and bird pancreatic PLA2, like dromedary [20], chicken [22] and ostrich [23] but less than those of the PLA2 from the pyloric ceca of starfish A. pectinifera [33], CDLA [28] and mSDPL [30] which had optimal temperatures around 50 °C.

The thermostability of SPLA2 was also investigated by measuring the residual activity after incubation of the pure enzyme at 70°C in buffer at different times (Figure 3B). In contrast to mSDPL, CDLA [28] and TPLA2 [21], which lose their full activities when incubated at 55 °C during a few minutes, PLA2 purified from stingray pancreas can tolerate the incubation at high temperature and maintained about 75% of its activity after 5 min incubation at 70 °C. Similar behavior was obtained with dromedary (DrPLA2) taken as model of mammal PLA2 when incubated under the same conditions at 70 °C (Figure 3B). However, marine PLA2 was found, less resistant against temperature than the ostrich PLA2 (OPLA2) taken as a model of bird PLA2. As shown in Figure 3A, pure OPLA2 maintained about 80% of its activity after 20 min incubation at 70 °C.

#### Effect of pH on the phospholipase activity and stability

The pH activity profile of the purified stingray phospholipase A_2 _is shown in Figure 3C. The pH-optimum of SPLA2 activity was similar to that of DrPLA2 and OPLA2 [20-23]**1760**. The maximal activity of SPLA2 was measured at pH 8.5 (Figure 3C).

Moreover, the pH stability (Figure 3D) showed that the purified SPLA2 was found to be active between pH 3 and 10 during 10 min of incubation. In contrast to OPLA2 which maintained more than 70% of its activity when incubated at pH 1.5 the pure SPLA2 is not stable at pH less than 3, (Figure 3D). However, the TPLA2 [21], mSDL [30] and CDPL [28] lose their full activity when incubated at pH less than 5 for few minutes.

#### Ca^2+ ^dependence

**Figure 4:**
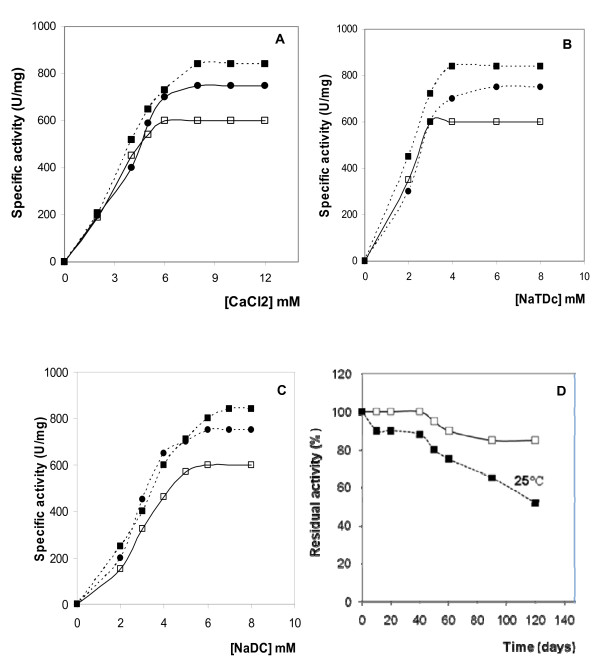
**Biochemical properties of SPLA2 compared with these of OPLA2 and DrPLA2**. (A) Effect of Ca^2+ ^concentration on SPLA2, OPLA2 and DrPLA2 activity. Enzyme activity was measured at various concentrations of Ca^2+ ^using PC as substrate at pH 8.5 and at 40°C in the presence of 4 mM NaTDC. The star symbol indicates the absence of phospholipase activity checked in the absence of CaCl_2 _and in the presence of 10 mM EDTA. (B) Effect of increasing concentration of NaTDC on SPLA2_, _OPLA2 and DrPLA2 activities. (C) Effect of increasing concentration of NaDC on SPLA2_, _OPLA2 and DrPLA2 activities. PLA2 activity was measured using PC emulsion as substrate at under standard conditions. (D) Long-term stability of SPLA2 stored in the refrigerator (5 °C) and at room temperature (25 °C). Assays were carried out under standard conditions as described in the text. SPLA2 (black circle); DrPLA2 (black square); OPLA2 (open square). All experiments were repeated at least three times

It is well established that Ca^2+ ^is essential for both, activity and binding of phospholipases to their substrate [34,35]. In order to investigate the effect of Ca^2+ ^on CDPL activity, we studied the variation of the PC emulsion hydrolysis rates by homogeneous SPLA2 in presence of various Ca^2+ ^concentrations (Figure 4A). For further comparison, we reported in the same Figure 4A the results obtained with DrPLA2 and OPLA2. Our results show that PLA2 activity could not be detected in the presence of chelator such as EDTA or EGTA when pure PC or egg yolk emulsion was used as substrate. The specific activity of SPLA2 increased to reach its maximum in the presence of 8 mM Ca^2+ ^using PC as substrate (figure 4A). Similar results were obtained with mSDPL [30] and CDPL [28]. This Ca^2+ ^concentration is also needed to activate mammal and bird pancreatic PLA2 [20-23].

#### Bile salts dependence

Several studies have provided evidence that bile salts are tensioactive agents ensuring in micellar form, the dispersion of the hydrolysis products and thus increase the hydrolysis rate. De Haas et al. (1970) [36] reported that micellar forms of the substrate were hydrolyzed by PLA2 at a much higher rate than molecularly dispersed substrates. In order to investigate the effect of bile salts on SPLA2 activity, the rate of hydrolysis of PC by SPLA2 with various concentrations of bile salts, at pH 8.5 and at 40 °C, was studied. As shown in figure 4C and 4D, sodium Taurodeoxycholate (NaTDC) and sodium deoxycholate (NaDC) were specifically required for SPLA2 activity. The maximum phospholipase activity was observed in the presence of 4 mM NaTDC or 6 mM NaDC. These observations corroborate with previous findings with mammals, bird pancreatic PLA2 [20-23].

#### Kinetic parameters

To determine the kinetic parameters of SPLA2, the rate of hydrolysis of different concentrations of PC were measured under optimal conditions (4 mM NaTDC, 8 mM_, _CaCl_2_, pH 8,5 and 40 °C). The Lineweaver-Burk curves were plotted (data not shown). From these fits, the substrate affinity constants (K_M_) and the turnover of the enzymatic reaction (k_cat_) were obtained and shown with the deduced catalytic efficiency (k_cat_/K_M_) in Table 3. For further comparison, we reported in the same Table 3 the kinetic parameters values obtained with DrPLA2, under the same conditions. From these values, one can say that SPLA2 hydrolyses the PC substrate more efficiently than SPLA2 since the ratio representing the catalytic efficiency (K_cat_/K_m_) is about 2 times higher with SPLA2 than with DrPLA2.

**Table 3:** Apparent kinetic parameters of SPLA2, and dromedary pancreatic phospholipase A_2 _(DrPLA2).

Phospholipase	V_max _(U/mg)	K_m _(mM)	K_cat _(s^−1^)	K_cat_/K_m _(mM^−1 ^s^−1^)
SPLA2	750	17	187	11

DrPLA2	600	22	140	6.36

#### Effects of organic solvents

Organic solvents can be advantageous in various industrial enzymatic processes. The use of organic solvents can increase the solubility of non-polar substrates, increase the thermal stability of enzymes, decrease water-dependent side reactions, or eliminate microbial contamination [37]. In this study, the SPLA2 showed high stability in the presence of water-miscible organic solvents, since it retained almost 100% activity after exposure, for 2 h at 25 °C, to 50% methanol, 50% ethanol, 50% 2-propanol, 50% acetonitrile or 50% acetone (Table 4). Addition of 50% ethanol or 50% acetonitrile to the pure SPLA2 caused a 12% immediate increase of the PLA2 activity in comparison to the control.

**Table 4:** stability of SPLA2 in organic solvents.

Organic solvent	Relative activity% (1 h)	Relative activity% (2 h)
Control	100 ± 0.5	100 ± 2.4

Acetone	95 ± 2.5	92 ± 2.2

Acetonitrile	105 ± 1.5	105 ± 2.3

Methanol	98 ± 2.4	95 ± 1.9

Ethanol	116 ± 2.7	100 ± 3.3

2-Propanol	97 ± 3.1	93 ±3.6

Pure SPLA2 was incubated in each organic solvent (50%) at 30 °C for 1 or 2 hours.

The experiments were conducted three times.

#### Long-term stability

In the course of the long-term stability experiment, the activity of SPLA2, which was stored at room temperature, did not decrease within the two first days (Figure 3). During the first week, activity did not drop below 90% of initial values. Later on, a continuous decrease was evident towards 50% of initial activity after 120 days. In contrast, the samples stored in the refrigerator maintained more than 90% of initial activity after 120 days. In conclusion, SPLA2 activity remained surprisingly stable up to 40 weeks, although SPLA2 was not maintained in a stability-enhancing medium, e.g. supplements of Ca^2+^, glycerol, or ammonium sulphate, but just in plain demineralized water.

## Materiels and methods

### Materials

Benzamidine was from Fluka (Buchs, Switzerland), bovine serum albumine (BSA), sodium deoxycholate (NaDC), sodium taurodeoxycholate (NaTDC), Triton X-100 (TX-100) and phosphatidylcholine (PC) were from Sigma Chemical (St. Louis, USA), acrylamide and bis-acrylamide electrophoresis grade were from BDH (Poole, UK). Marker proteins and the chromatography supports, used for PLA2 purification: Sephadex G-50, Mono-S, Mono-Q were Pharmacia (Uppsala, Sweden).). PVDF membrane and protein sequencer Procise 492 equipped with 140 C HPLC system purchased from Applied Biosystems (Roissy, France). C-8 reverse-phase eurospher 100 column was from Knauer (Germany). pH-stat was from Metrohm (Herisau, Switzerland).

### Pancreas collections

Stingrays (Dasyatis pastinaca) pancreases were collected from a local fish market (Sfax, Tunisia) and stored at -20°C.

### Determination of phospholipase activity

The stingray PLA2 activity was measured titrimetrically at pH 8.5 and at 40 °C with a pH-stat, under the optimum conditions, using purified egg PC or a crude egg yolk emulsions as substrate in the presence of 4 mM NaTDC and 8 mM CaCl_2 _[38]. Some assays were performed with NaDC. One unit of phospholipase activity was defined as 1 μmole of fatty acid liberated under standard conditions.

### Effects of temperature and pH on SPLA2 stability

In order to check the thermal stability of SPLA2, homogeneous enzyme was incubated successively at 70 °C for different durations. The pH stability of SPLA2 was studied at room temperature during 30 min using the following buffers: 50 mM sodium acetate buffer (pH 4-6), 50 mM potassium phosphate buffer (pH 6-8), 50 mM Tris-HCl buffer (pH 7-10). After each incubation, residual phospholipase activity was measured after centrifugation of the sample, under optimal conditions.

### Determination of protein concentration

Protein concentration was determined as described by Bradford (1976) using BSA (E^1%^_1 cm _= 6.7) as reference [33].

### Oligosaccharide content

The presence of glycan chains in the purified cofactors was checked by the anthrone-sulfuric acid method using glucose as a standard [39]. One milliliter of each pure SPLA2 (1 mg/ml in Tris-HCl buffer) was mixed with 4 ml of distilled water in screw cap type culture tube. The tube was then placed on ice to cool. Then, we added 10 ml of cold anthrone reagent (0.2 g in 100 ml concentrated H_2_SO_4_) prepared fresh daily. After mixing, we placed a marble on top of the tube to prevent evaporation and we incubated in a boiling water bath during 16 min, afterwards, the tube was cooled on ice for 2-3 min then at room temperature for 5-10 min. Finally, we read the absorbance at 620 nm against a reagent blank. The rate of glycosylation is calculated on the basis of percentage by weight.

### Alkylation of cysteine residues

The alkylation of cysteine residues of phospholipase was realized as described by Okazaki et al. (1985) [40]. Hundred picomoles of SPLA2 in 1 ml of 10 mM Tris-HCl, pH 8 were denatured in 185 μl of 8 M guanidine hydrochloride, 65 μl of 1 M Tris-HCl, 4 mM EDTA (pH 8.5) and 80 mM DTT for 30 min at 60 °C. S-Pyridylethylation of cysteine residues of protein was performed by adding 4 μl of vinyl pyridine and incubation at 25 °C for 3 h. The modified enzyme was dialyzed against water for N-terminal sequencing.

### Analytical methods

Analytical polyacrylamide gel electrophoresis of proteins in the presence of sodium dodecyl sulfate (SDS-PAGE) was performed by the method of Laemmli (1970) [41]. The proteins were stained with Coomassie brilliant blue.

### Amino acid sequencing

For N-terminal sequencing, the purified enzyme was blotted (60 min, 50 mA, 4 °C) onto a PVDF (polyvinylidene difluoride) membrane (Applied Biosystems, ProBlotTM) in 20 mM CAPS buffer (pH 11) containing 10% methanol using a mini trans-blot cell (BioRad, Hercules, USA). The N-terminal sequence was determined by automated Edman's degradation, using an Applied Biosystems Protein Sequencer Procise 492 equipped with 140 C HPLC system (Roissy, France) [42].

## Conclusion

Described here is the purification and the characterization of a new phospholipase A2 from stingray pancreas. This phospholipase has several advantageous features for industrial applications. Stability of SPLA2 in the presence of organic solvents, and its tolerance to high temperatures, basic and acidic pH, makes it a good candidate for application in food industry to treat phospholipid-rich industrial effluents, or to synthesize useful chemical compounds.

## Abbreviations

AA: Arachidonic acid; DrPLA2: dromedary phospholipase A2; k_cat_: the turnover of the enzymatic reaction; K_M_: substrate affinity constants; NaTDC: sodium taurodeoxycholate; OPLA2: ostrich phospholipase A2; PLA2: phospholipase A2; SPLA2: stingray phospholipase A2, sPLA2-IB: secreted pancreatic phospholipase A2;

## Competing interests

The authors declare that they have no competing interests.

## Authors' contributions

ABB and AK carried out all the studies, analyzed the data and drafted the manuscript. EB helped with the analysis of the data. YG helped with the discussion of the data and the correction of the manuscript. YBA participated in the study design and helped to draft the manuscript. All authors have read and approved the final manuscript.
